# Sex Differences in Infective Endocarditis: A Systematic Review

**DOI:** 10.7759/cureus.49815

**Published:** 2023-12-02

**Authors:** Ethan Slouha, Hanin Al-Geizi, Brandon R Albalat, Venkata Sathya Burle, Lucy A Clunes, Theofanis F Kollias

**Affiliations:** 1 Anatomical Sciences, St. George's University School of Medicine, St. George's, GRD; 2 Pharmacology, St. George's University School of Medicine, St. George's, GRD; 3 Pharmacology, St. George's University, St. George's, GRD; 4 Microbiology, Immunology and Pharmacology, St. George's University School of Medicine, St. George's, GRD

**Keywords:** cardiology, microbiology, gender differences, sex differences, infective endocarditis

## Abstract

Despite the advancement in medicine, there is still a lack of understanding of the sex disparities in disease onset, progression, treatment, and outcome. In some life-threatening acute conditions, despite most patients with these illnesses being males, females have a significantly higher chance of mortality. This can be due to the differences in disease progression or healthcare disparities in managing the illness between the sexes. Treatment of illnesses tends to be more conservative for women without an explanation, but this disparity is due to the healthcare provider. Infective endocarditis (IE) is an acute life-threatening condition where bacteria latch onto and seed damaged endocardium, with some preliminary information reporting differences between the sexes. This paper aims to evaluate the sex disparities in the incidence, age, comorbidities, etiology, risk factors, manifestations, treatment, and outcomes of IE.

From 2003-2023, 21584 articles were found that focused on the sex differences in IE and, through PRISMA guidelines, were narrowed down to 34 publications. There are significant differences between the sexes in IE, such as a significantly higher incidence of IE in males, who also tend to be older and have their native aortic valves involved, compared to younger females who have their mitral valve involved. Comorbidities also vary between the sexes; females tend to have atrial fibrillation, chronic kidney disease, psychiatric disorders, and taking immunosuppressants compared to males who suffer from chronic liver disease, underlying valve disease, and peripheral artery disease, contributing to the ease of developing IE. While the most common microorganism leading to IE is *Staphylococcus aureus,* females were more likely to have culture-negative IE, and men were more likely to be infected with *Streptococcus viridans. *Major manifestations in IE are fever and vegetation along the closure of the valves in the heart, where females were more likely to have vegetation on the mitral and aortic valves. At the same time, males were more likely to have it on the tricuspid valve. On par with sex disparities in health, females usually took longer to seek medical help than males despite the advancement of symptoms and deterioration. Females were also treated conservatively through antibiotic management, whereas males were more likely to advance to surgical treatment, leading to a longer hospital stay. While there was no true difference in the in-hospital mortality rate, the 30-day and 1-year mortality were significantly increased in females.

These differences provide a range of starting points for various research to further educate physicians on sex disparities, such as why males have a higher incidence of infective endocarditis and determining whether it’s hormones and basic metabolites, possibly limiting those who develop the infection. Another important point is treating females with IE; the antibiotic doses are standard, but whether they advance to surgical treatment is mostly up to the provider. Some providers deny surgical treatment despite all indications, but it could also be females denying surgery as they tend to leave against medical advice. This review is crucial in developing the next steps to sex disparity in IE, which may lead to better outcomes for males and females.

## Introduction and background

Sex disparities in diseases

Males and females differ significantly in aspects of health, ranging from origins to mechanisms to how their body reacts to outcomes, and even though this is out of their control, how they are treated for the disease. Differences are based on genetic vulnerability, hormonal and reproductive factors, and physiological differences [1, 2]. Females tend to live up to 7 years longer than males, which has been loosely associated with the finite stage of reproductive functioning [1, 2]. Males suffer from major acute illnesses for short periods [2]. Males tend to have acute diseases as they are more likely to smoke, drink alcohol, and use drugs more frequently, leading to more significant health complications [2]. Females are more likely to develop osteoporosis, arthritis, hypertension, and diabetes compared to men [2]. Factors that can lead to the development of these chronic conditions have been associated with females, on average, having lower educational status in some countries and the tendency to live alone when older [2]. Long-term effects of conditions in females may also be influenced by the fact that females are more likely to suffer from anxiety about the condition and treatment and may not monitor their progress as accurately [1, 2].

Frameworks concerning the treatment methods of most diseases used to be based on males before the 1950s. However, since then, studies on treatment methods have been centered around both sexes, changing treatment methods to some extent [2]. In developing countries, compared to women, men actively seek treatment more often in health services [2]. Whereas women were more likely to use alternative therapies or self-treat, this may be due to their role in domestic spheres [2]. Even when treatments have been compared, females are more likely to be treated inferiorly at hospitals, such as providers believing they are over-dramatizing symptoms or blamed for coming in late concerning symptoms they are experiencing [2]. The insensitive treatment providers tend to give towards females has been identified as a contributor to avoiding professional help [2]. An example of treatment differences is that females who suffer from atrial fibrillation are less likely to receive anticoagulants such as warfarin despite having a greater risk for stroke [3]. Also, females are less likely to receive a guideline-based diagnosis and less invasive treatment; however, possibly due to the underlying differences in sex, females still tend to have better outcomes [3]. When treatment is allocated appropriately to both sexes, males tend to be less compliant with the treatment that has been recommended [2].

Even when both sexes are diagnosed with the same conditions, males are more likely to have a higher mortality than women [2]. Females were more likely to have a longer recovery, but this could be due to delays in getting treatment [2]. Females also frequently return to work before fully recovering from treatment, which can affect their outcomes [2]. Males are the opposite, possibly because their support network encourages them to come in sooner for treatment and to take their time in recovery [2]. Males frequently receive post-treatment care from their partner, which improves their outcome [2]. Also, as a disease progresses, females tend to have less “will to live” about detrimental disorders and would not choose to go through life-sustaining care [2]. Ultimately, it seems that females' prominent advantage concerning diseases is their physiological build to allow pregnancy, which means the disease courses are less impactful than males [2]. Whereas with males, their significant advantage may be lower levels of role stress, lower societal demands, and role conflict [2].

Infective endocarditis

Infective endocarditis (IE) is a rare condition requiring multiple independent factors: modifications to the surfaces of cardiac valves and bacteremia with an organism that can adhere to damaged endothelium and seed the infection into the bloodstream [4]. Injury to the endocardium may result from a turbulent flow generated by damaged valves or direct trauma caused by either the presence of an electrode or a catheter [5]. Cases of IE are greater amongst individuals with highly turbulent lesions, such as individuals with minor ventricular septal defects or valvular stenosis [5, 6]. High-pressure flows typically create increased localized damage to the endothelium, which can result in a site for bacterial vegetation to form [5]. Commonly involved valves in IE include the native mitral valve and native aortic valve [6]. Mitral valve prolapse comprises approximately 20% of IE cases [6]. Patients with a bicuspid aortic valve, syphilis, or Marfan’s syndrome have an increased risk of developing IE as well [6].

Once bacteria have entered the bloodstream, a host of manifestations develop fever, night sweats, aching joints, Osler nodes, flu-like symptoms, chest pain, fatigue, Janeway lesions, and vegetation [7]. Hemodynamics plays a significant role in forming vegetation over the ventricular surface of the aortic valve and the atrial surface of the mitral valve [5]. Damage to the endocardium serves as an area for platelet aggregation and activation of the clotting cascade, which gives rise to sterile, non-bacterial thrombotic forms of vegetation [4, 5]. Vegetations are typically localized downstream from a regurgitant flow, which favors the theory of decreased perfusion, resulting in endothelial injury where bacteria can form vegetation [5]. Once vegetation forms, bacteremia can result in bacterial colonization of the vegetation [5]. Further complications such as heart failure, stroke, abscesses, kidney damage, and splenomegaly can occur due to IE, which persists as lifelong complications [7].

Risk factors for developing IE include advanced age, diabetes, use of steroids, pacemaker, poor dental health, and residual valve injury [6, 7]. Recently, IE has been observed to develop more frequently through healthcare-associated procedures such as early prosthetic valve replacement, hemodialysis, hospitalizations, cardiac operative procedures, or catheterization. Community-acquired cases of IE occurrences have not changed and typically arise in the immunosuppressed population, those with poor dentition, IV drug users, those with Rheumatic heart disease, and those with degenerative valve disease [5]. In IV drug users, IE develops as the repetitive injection leads to the degeneration of valves by particulate matter, such as *Staphylococcus aureus* and *Staphylococcus epidermidis*, that reside on natural skin flora that may be co-injected with the drugs [5]. Even with the injury to the endocardium and bacteremia, pathogenesis requires a virulent organism capable of adhering to and promoting platelet-fibrin deposition [5]. The majority of all cases of IE arise from *Staphylococci, Enterococci*, and *Streptococci* organisms, with 50% of all healthcare-associated IE cases attributed to *Staphylococcus aureus* [5]. Fungal organisms causing IE account for only 1% of all patients and are usually a result of complications of systemic aspergillus and candida infections in those immunocompromised [5]. It is hypothesized that these virulent organisms need to expand the platelet-fibrin deposit so that it will protect the organism from the host's immune system [5].

The treatment approach for IE must be multidisciplinary, involving cardiologists, cardiothoracic surgeons, and infectious disease personnel [8]. Treatment usually begins with antibiotics to try and control the bacteremia, and these are generally given for 2-6 weeks [9]. Initially, patients are prescribed broad-spectrum empiric antibiotics, and once cultures come back, they are switched to a specific antibiotic or two [9]. Specifically for patients with gram-positive-based IE, a monotherapy treatment with vancomycin is usually given [9]. At the same time, individuals need to be placed on antithrombotics due to increased clotting associated with other comorbidities that patients typically have [8]. However, this additional therapy is strongly evaluated based on benefits and risks, with the benefit being a reduction in the complications of an embolic stroke [8]. Surgical treatment is considered if antibiotics do not work efficiently or the patient deteriorates rapidly on admission. Surgery performed for IE is through valve removal or pacemaker placement and surgical debridement of vegetation coated with bacteria, which is delayed in patients with major cerebrovascular complications [8]. Understanding and analyzing IE is crucial because the mortality rates significantly increase as time progresses, with 24% mortality at 1 year, 42% at 5 years, 50% at 10 years, and 56% at 20 years [10]. Also, once an individual has IE, they are at an even greater risk of developing IE again. Antibiotic prophylaxis usually prevents this and limits predisposing factors such as frequent IV insertions [8]. However, like many conditions such as myocardial infarction and atrial fibrillation, IE may display sex differences such as source, etiology, course, treatment, and outcome.

Aim

This paper aims to elucidate any difference in the development and variations in the treatment and outcomes of IE. To our knowledge, this is the first systematic review evaluating the differences in IE between the sexes. The incidence of IE between sexes will be assessed, including age, comorbidities, and associated risk factors compared with IE overall. The etiologies will be compared between the sexes concerning the main microorganism for IE. Notably, the manifestations will be equated due to the differences in hormones between the sexes. Equally as important is the comparison of treatment because, as mentioned, there’s a difference in how females and males are evaluated and treated by providers, and this could influence the outcome of IE in both individuals. Additionally, articles focused on the cost of care between the sexes, and the results were compared.

## Review

Methods

All searches and composition of the articles strictly adhered to the PRISMA guideline. They included a meticulous and extensive literature search of PubMed, ProQuest, and ScienceDirect catalog from January 1, 2003, to November 1, 2023. The search keyword consisted of ‘Sex difference in infective endocarditis’. The search concentrated on peer-reviewed experimental and observational publications only containing the keyword. Publications published before 2003 were not written in English, and those duplicates were excluded from the screening process. Once publications were gathered, four independent co-authors assessed the information and compiled the results. These publications were evaluated based on the abstracts, title, study type, and full-text accessibility. This initial analysis of the three catalogs resulted in 21584 publications. Keyword specifics and the information gathered from the abstract further narrowed down the selected publications. A total of 34 publications were found that covered the scope of this paper according to the following criteria:

Inclusion Criteria

The criteria for inclusion included publications published between 2003 and 2023, focused on sex differences in IE, experimental studies, observational studies conducted on humans, written in English, and full-text.

Exclusion Criteria

**Figure 1:**
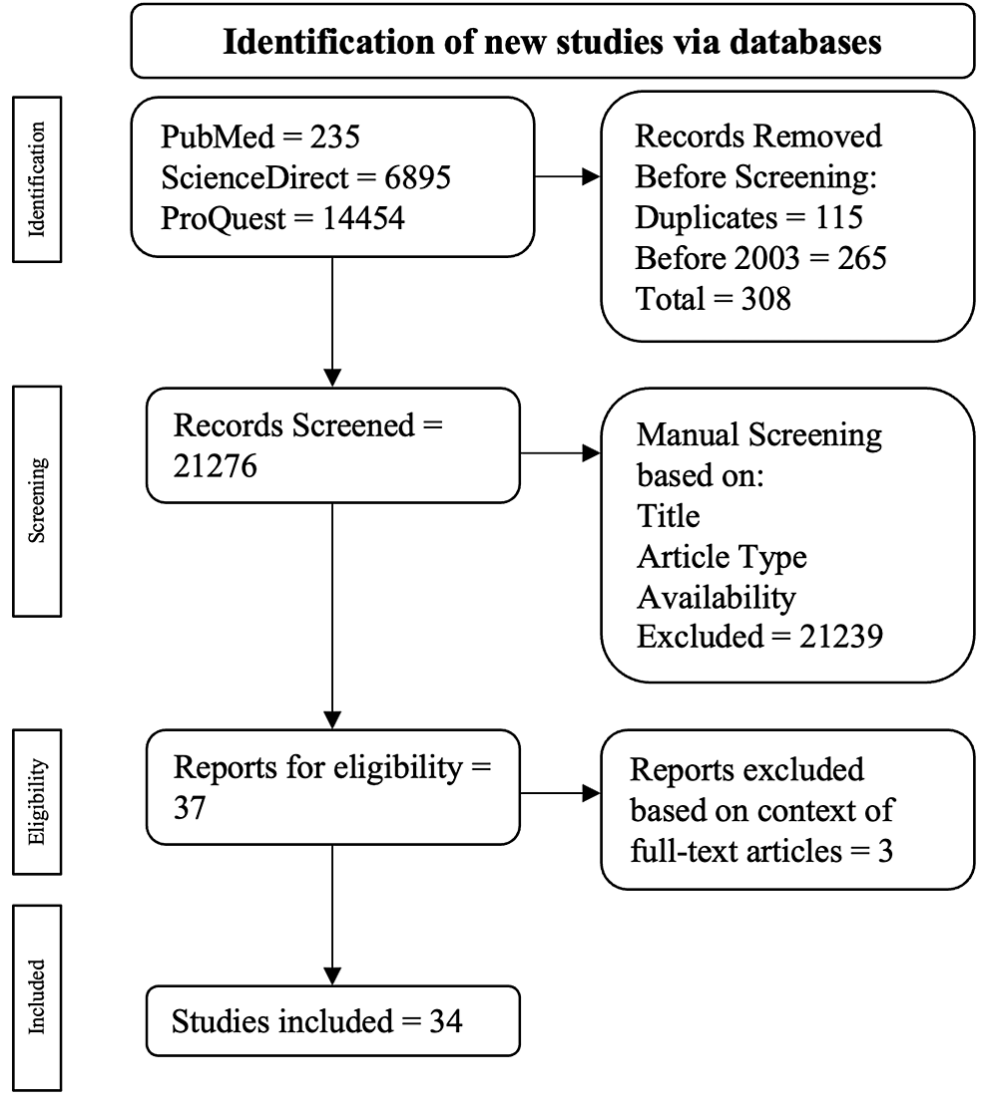
Visual representation of the inclusion and exclusion criteria process The pathway used for the inclusion and exclusion criteria came from the PRISMA review [11] PRISMA - Preferred Reporting Items for Systematic Reviews and Meta-Analyses

The criteria for exclusion ruled out meta-analyses, narrative reviews, and case reports/series. All duplicates and articles that were not full text were excluded as well. This article's inclusion and exclusion procedure is drawn out in Figure 1.

Bias

All publications included in this study were viewed for bias. The overall bias in this paper is minimal, and all methods were explained appropriately. A good amount of studies extrapolated data from global data banks as well. Each article was evaluated for bias using the GRADE (grading of recommendation, assessments, development, and evaluation) scale and was found to be moderate. The GRADE tool was used in this article to evaluate the risk of bias in each paper, weighing flaws like imprecision, indirectness, and publications.

Results

Twenty-one thousand five-hundred and eighty-four publications were found: 235 were from PubMed, 6895 were from ScienceDirect, and 14454 were from ProQuest. Among the exclusions, 115 were duplications, and 265 were published before 2003. This resulted in 308 publications being excluded during the programmed screening process, leading to 21276 publications for manual screening. Publications were manually assessed through the criteria mentioned previously, resulting in 37 articles being checked for eligibility through full-text analysis. Ultimately, 34 articles were used.

IE occurs in native or prosthetic valves, usually when there is damage to the endothelium present. While its incident rate is low, its mortality is high, and there are some significant differences concerning its course between males and females. Males have a significant incidence of IE, specifically with the native aortic valve, compared to females. Regarding age, females tended to be significantly older than males at diagnosis. Females were also more likely to undergo hemodialysis, immunosuppressant therapy, or psychiatric disorders than men who likely had chronic liver disease, previous coronary artery bypass graft (CABG), or peripheral artery disease. The number one microorganism causing IE did not differ between the sexes, which was *Staphylococcus aureus*, but there is a variation in the other common microorganisms. Females were more likely to have culture-negative endocarditis and be infected with gram-negative bacteria. Males, however, were more likely to be infected with *Streptococcus viridans*. Concerning manifestations, females were more likely to develop vegetation on the mitral and aortic valves and an increase in complication rate. At the same time, males tended to present earlier to the ER with vegetation on their tricuspid valves and present with significant valvular regurgitation. Regarding treatment, females were less likely to receive surgical treatment and were primarily treated conservatively with antibiotics, even when presenting with surgery indications. Long-term mortality also varied between the sexes, as females tended to have a higher mortality rate as time progressed. Information involving the results and conclusions of articles used for analysis in this paper can be found in Table 1.

**Table 1:** Summary of articles used in this review according to PRISMA guidelines [11] IE - Infective Endocarditis; CRP - C-Reactive Protein; ASIRs - Age-Standardized and Sex-Stratified Incidence Rates; ASMRs - Age-Standardized and Sex-Stratified Mortality Rates; ASMIRs - Age-Standardized and Sex-stratified Mortality-to-Incidence Ratios; T2DM - Type 2 Diabetes Mellitus; SAVR - Surgical Aortic Valve Replacement; NIE - Nosocomial Infective Endocarditis; CIE - Community-Acquired Infective Endocarditis; AVR - Aortic Valve Replacement; MVR - Mitral Valve Replacement; TAVR - Transcatheter Aortic Valve Replacement; IV - Intravenous; PV - Prosthetic Valve; PRISMA - Preferred Reporting Items for Systematic Reviews and Meta-Analyses

	Author	Country	Design & Study Population	Findings	Conclusion
1	Aksoy et al., 2017 [15]	USA	Prospective Cohort Study (n = 439)	T2DM, immunosuppression, and hemodialysis occurred more frequently in female patients with IE, and these patients were less likely to undergo surgery. New conduction abnormalities and intracardiac abscesses were more common in males.	Differences in the treatment and outcomes of IE between sexes result in co- and pre-existing conditions such as hemodialysis, diabetes mellitus, and chronic immunosuppression.
2	Bhandari et al., 2022 [32]	USA	Retrospective Cohort Study (n = 780)	IE incident was equal by sex. More females tended to have tricuspid valve endocarditis, were younger than males, and were discharged against medical advice.	There is a significantly higher occurrence of IE in younger females, and they were more likely to leave against medical advice when looking at a state with a high incidence of drug use.
3	Friedrich et al., 2022 [23]	Germany	Retrospective Observational Study (n = 413)	Females exhibited a higher frequency of the mitral valve, while males presented more with previous surgically treated IE, coronary heart disease, and isolated aortic valve endocarditis. Intraoperative blood transfusions occurred more frequently among females, whereas postoperative transfusions were more prevalent among males. Males had a longer postoperative stay. The female sex was an independent predictor for 30-day mortality after surgical treatment of IE and showed significantly higher 30-day mortality. There was no sex-specific difference in late survival.	Females showed a higher early mortality, and female sex was an independent predictor for 30-day mortality. There was no significant difference during follow-up regarding mortality. Differences specific to each sex were observed in the risk factors associated with both early and late mortality, such as elevated preoperative CRP levels were more prevalent among females, whereas culture-negative infective endocarditis was more commonly seen in males.
4	Hammond-Haley et al., 2023 [33]	United Kingdom	Observational Study (n = 19 countries)	ASIRs of IE increased in both sexes in 19 countries between 1990 and 2019 but were higher in males than females. ASMRs were higher in females in Italy. ASMIRs were higher in females.	The incidence and mortality of IE have increased over the last 30 years. *Staphylococcus aureus* has become the primary agent responsible for IE, frequently impacting susceptible or older patient groups.
5	Jensen et al., 2021 [24]	Denmark	Population study (n = 8,675)	The first time IE during 1997-2017 showed an increase in the incidence rate in both males and females. The incidence rate for patients without prior prosthetic heart valves or a cardiac implantable electronic device increased. The incidence rate ratio adjusting for age groups, sex, and diabetes increased in 2017.	The occurrence of IE increased by more than twofold. This rise was particularly noticeable among male and older patients.
6	Lopez-de-Andres et al., 2022 [43]	Spain	Retrospective Cohort Study (n = 9,958)	The incidence of IE significantly increased in mostly males with T2DM. When adjusting for age, the incidence of IE was considerably higher in individuals with T2DM of both sexes compared to those without T2DM. In-hospital mortality was higher among T2DM females. The existence of T2DM was not associated with in-hospital mortality in both sexes.	T2DM is linked to an increased occurrence of hospitalization due to IE. T2DM patients who had encountered IE varied based on sex, with males having higher incidence rates and lower in-hospital rates compared to females. It was observed that T2DM did not increase in-hospital mortality for either males or females diagnosed with IE.
7	Mettler et al., 2023 [34]	United States	Population study	During 1990-2019, the ASIR of IE in the United States has increased mostly in males 55+ years old. There was a rise in the occurrence of IE between 2015 and 2019 in all age groups, with the most notable increase observed in individuals aged 5 to 19. In the United States, the age-adjusted mortality linked to IE significantly increased.	The incidence and mortality of IE increased from 1990-2019. Despite the general rise, notable variations exist across geographical regions, sexes, and age categories.
8	Myllykangas et al., 2020 [45]	Finland	Retrospective Cohort Study (n = 5,628)	The incidence of IE was higher among males with biological valve prostheses than females. While aortic valve surgery was more frequent in males during the 1–5-year period, this tendency did not persist at the 10-year mark.	There was a higher risk in males encountering bleeding, requiring early re-surgery after SAVR, and developing IE when using biological prostheses.
9	Ortega-Loubon et al., 2019 [25]	Spain	Retrospective Cohort Study (n = 28,475)	Most individuals diagnosed with NIE were male, older (63.8 vs. 60.8), had a more significant history of implanted cardiac devices, and had a higher mortality rate. *Staphylococcus aureus* was the most found microorganism in both NIE and CIE.	The general occurrence of NIE has risen while CIE has decreased. Individuals affected by NIE showed a strong correlation with increased mortality and a poorer prognosis than CIE patients.
10	Ostergaard et al., 2018 [13]	Denmark	Retrospective Cohort Study (n = 18,041)	Patients who underwent left-sided heart valve replacement (mitral or aortic valve) between 1996 and 2015 are at high risk for IE. The cumulative risk of IE at a 10-year follow-up increased for both MVR and AVR. Factors such as male sex and bioprosthetic valve were associated with increased risk of IE for both MVR and AVR.	Following left-sided heart valve replacement, the occurrence of IE is relatively frequent, affecting approximately 1/20 patients over a 10-year period.
11	Panagides et al., 2022 [38]	Canada	Retrospective Cohort Study (n = 579)	Sexual disparities in IE following TAVR were observed. Female patients tended to be older than males and displayed a lesser burden of comorbidities. Culture-negative IE was more prevalent among females. The mortality rate during the initial IE hospitalization was similar between the two groups, yet females exhibited a higher mortality rate at the 2-year follow-up.	There were no significant sex differences in the clinical attributes and treatment approaches for IE following TAVR. Females were linked to an elevated risk of mortality at the 2-year mark.
12	Dohmen et al., 2016 [42]	Germany	Prospective Cohort Study (n = 755)	The early mortality rate was significantly higher in females. In male patients, variables associated with overall mortality were insulin-dependent diabetes mellitus, age, previous cardiac surgery, and low ejection fraction preoperatively. Focus identification, preoperative dialysis, involvement of multiple valves, and age were risk factors for late mortality in females.	There are distinct differences in mortality as well as the risk of mortality in patients with IE, including varying early and long-term outcomes.
13	Elamragy et al., 2020 [21]	Egypt	Retrospective Cohort Study (n = 398)	Males were significantly older than females, but malignancy and recent procedures were more common in females, while IV drug abuse and chronic liver disease were more common in males. Rheumatic valvular disease was more common in females. There were no significant differences in major complications such as mortality and fulminant sepsis.	Males are more likely to develop IE, while females are significantly younger. IV drug use leading to IE was more common in males, who also had a higher occurrence of IE in structurally normal hearts. Females had a higher rate of overall complications.
14	Afshar et al., 2023 [14]	Iran	Cross-Sectional Study (n = 75)	There were 75 patients, of which 34.7% were females. In general, females had a higher chance of having T2DM, hypertension, and hypercholesterolemia. On the other hand, males had a greater chance of having a smoking history. Females, in general, had worse outcomes following mitral valve repair.	The majority of adverse events, such as mortality, cardiac and cerebrovascular events, reoperation, and treatment failures, were greater amongst females than males. The poor outcomes seen in females might be linked to the fact that females tend to have a more adverse risk profile.
15	Polishchuk et al., 2021 [41]	Israel	Retrospective study (n = 214)	Coagulase-negative *Staphylococcus* occurred more frequently in males, while culture-negative endocarditis occurred more in females. There was no difference in the primary outcome between the sexes, but the all-cause mortality of females was significantly higher.	Sex has an important role in the microbial profile and outcome of patients who have IE.
16	Sambola et al., 2010 [26]	Spain	Prospective Cohort Study (n = 271)	Females tended to be older than males, had mitral valve involvement, and were taking immunosuppressants. There were no sex differences in regurgitation severity, Charlson index, major complications, or culprit pathogens. Females, however, were less likely to undergo surgery, and mortality was higher in females in-hospital and at 1 year.	Females who have IE were older than males but showed similar comorbidities, as well as underwent surgical procedures less frequently, possibly leading to a worse prognosis.
17	Sevilla et al., 2010 [39]	Spain	Retrospective Cohort Study (n = 621)	Females tended to be older and had T2DM, prosthetic valve endocarditis, rheumatic cardiopathy, and NIE. Mechanical prosthetic mitral valve endocarditis was more common in females while males were associated with native aortic endocarditis. *Staphylococcus aureus* was the most common between the sexes.	Clinical characteristics, outcomes, and treatment approaches were similar between the sexes despite epidemiological, microbiological, and echocardiographic differences.
18	De Miguel-Yanes et al., 2022 [20]	Spain	Retrospective Cohort Study (n = 10,459)	Poisson regression analysis of the Spanish National Discharge data between 2016 and 2020 concerning age-adjusted time trends yielded a two-fold increased crude rate of IE incidence in males versus females. When rates were adjusted for potential confounding variables, females were found to have a higher rate of IE-related mortality. Females were diagnosed at later ages and presented with fewer comorbidities as stratified with the Charleston Comorbidity Index.	The incidence rate of IE was higher in males, whilst the rate of IE-related mortality was higher in females. Females had a lower rate of invasive cardiac procedures concerning IE-induced hospital admission.
19	Chew et al., 2021 [36]	USA	Retrospective Cohort Study (n = 18702)	More hospitalized females with IE die less than males. Females were hospitalized shorter times and had a lower overall hospital expense bill. Females were significantly less likely to undergo surgery as treatment.	There were notable differences in IE survival, management, and cost between males and females, with females being disadvantaged. This is likely due to sex-based bias.
20	Bezerra et al., 2021 [19]	Brazil	Cross-Sectional Retrospective Study (n = 211)	48 years old was the median age of the sampled population; males comprised 70.6% of cases. The most common pathological cause of IE was noted to be caused by *Staphylococcus spp*. Native aortic valve was infected more often in males than in females.	Of all the 211 cases included, it seems that age had the largest influence on deaths in patients with IE. The statistics plateaued with infection rates. The most common cause of IE in males was *Staphylococci spp*. and typically involved the native aortic valve.
21	Bell and Adegboye, 2023 [18]	Australia	Cross-sectional Retrospective Study (n = 18,044)	Factors that impact the outcomes of patients with IE include differences in sex, age, and place of birth. Females have an overall higher risk of death from IE than males. Death rates of IE were highest amongst patients who were older than 60 years old. Despite high volumes of IE admissions, the ICUs saw few patients. Amongst those patients that were admitted to the ICU, typically half of them would die.	Some of the most important predictors of increased mortality or severe disease were age, sex, and place of birth of the patient.
22	Becher et al., 2022 [17]	Germany	Contemporary Cohort (n = 86,469)	The incidence of IE in Germany increased between 2007 and 2019. *Staphylococcus *and *Streptococcus* with an increased presence and increased in-hospital mortality. Blood culture negative has decreased prevalence. Risk factors for in-hospital mortality are female sex, age, CV comorbidities, need for dialysis, sepsis, or invasive ventilation.	The incidence of IE was noted to have increased over time. In-hospital mortality was noted to have remained high. *Staphylococcus spp.* and gram-negative organisms causing bacteremia were associated with a greater likelihood of mortality within the hospital.
23	Sousa et al., 2021 [27]	Portugal	Retrospective Cohort Study (n = 7574)	Men composed 56.9%, and women composed 43.1% of hospitalized IE cases. Women were found to be older than men, less frequently had additional cardiovascular comorbidities, and presented with atrial fibrillation and arterial hypertension more frequently. Women had higher rates of acute heart failure, while men had higher rates of sepsis and acute renal failure. Women had a greater post-operative mortality and were found to have cardiac surgery less.	When hospitalized with IE, the female sex was noted as a risk factor for increased postoperative mortality; however, the overall rates of in-hospital mortality were concluded to not be affected by the patient's sex.
24	Weber et al., 2019 [31]	Germany	Retrospective Cohort Study (n = 305)	Out of all the patients, 75.1% of patients were male, and 24.9% were females and had undergone surgery. Infection of the mitral valve was seen more in females, and *Staphylococcus aureus* was discovered more in females than in men. Females were noted to have higher rates of both 30-day and 1-year mortality.	The female sex was found to have a more severe presentation of IE and greater 30-day and 1-year rates of mortality. The sex of the patient was found not to affect the clinical outcomes of IE.
25	Bansal et al., 2021 [16]	USA	Retrospective Cohort Study (n = 81,942)	Females were less likely to have cardiac valve replacements, including mitral, aortic, and combined mitral and aortic. However, still had relatively similar rates of death within the hospital as males. In both males and females, in-hospital mortality dropped. Amongst the patients who underwent valve replacement surgery, the mortality rate in-hospital was greater amongst females.	Although there has been an increase in usage of valve surgery for IE in both males and females alike with improvements in mortality trends, there is still a treatment bias where females still go undertreated. Therefore, in-hospital mortality rates of females were still higher than males.
26	Anderson et al., 2004 [44]	US	Case-Control Study (n = 41)	Patients who have a prosthetic valve and are co-infected with *Enterococcus* are at a greater risk of developing IE. Sex, race, age, polymicrobial infections, and community-acquired infections had no role in risk for IE.	Enterococcal IE can no longer be considered solely a unimicrobial community-acquired cause of IE in Caucasian males. Rather, data suggest that patients who have a PV and are infected with *Enterococcus fecalis* tend to have a higher risk of developing IE.
27	Ahtela et al., 2021 [35]	Finland	Retrospective Cohort Study (n = 2166)	Of all the patients in the study, 67.8% were males. Females were typically older than males. The median length of stay was 20 days among males and 18 days among females. Overall, in-hospital rates of mortality were 10% between both sexes. The 5 and 10-year mark for mortality rate was greater amongst females.	Males had overall longer in-hospital stays because of IE when compared to females. 5 and 10-year rates of mortality were higher amongst females. There were no changes over time in the mortality rate from IE or LOS.
28	Ahtela et al., 2019 [12]	Finland	Retrospective Cohort Study (n = 2611)	Of all cases of IE, 68.2% were males with an average age of 60. Females were significantly older than male patients. Males had a significantly greater risk of developing IE when compared to females. Between both males and females, mortality was similar. Mortality increased with age and remained similar between sexes.	There has been a notable increase in cases of IE amongst younger individuals in Finland. Middle-aged males are at the greatest risk of developing IE compared to females. 30-day mortality stayed stable at 11%. It remained similar between males and females and increased with age.
29	Curlier et al., 2014 [37]	France	Retrospective Cohort Study (n = 620)	Females tended to be older than males, were likely on hemodialysis, and had mitral valve IE. Females were less likely to develop septic shock or undergo early valve surgery. However, between the sexes, in-hospital mortality rates were comparable.	Females were more likely to have mitral valve IE, be older, and undergo hemodialysis while being less likely to undergo early valve surgery. Despite these differences, the in-hospital mortality rate was comparable between the sexes.
30	Erichsen et al., 2016 [22]	Denmark	Retrospective Cohort Study (n = 5,486)	There was a higher incidence in males with IE, who tended to be younger than 3 years on average. The age at which IE is diagnosed has decreased while the overall incidence has increased, which was more pronounced in males.	Males were more likely to develop IE and be younger. The overall incidence of IE is increasing and more prominent in men.
31	Sunder et al., 2019 [28]	France	Retrospective Cohort Study (n = 6,235)	The incidence of IE was 63 cases/million residents, with *Staphylococci *and *Streptococci* being the most common microorganisms. The in-hospital mortality was around 21% and was associated with renal failure, chronic liver disease, > 70 years of age, and strokes.	The most common microorganisms leading to IE were Staphylococci and Streptococci, with an in-hospital mortality rate of 21%.
32	Tariq et al., 2004 [29]	Pakistan	Prospective Cohort Study (n = 66)	The ratio between males and females was 2:1, with a median age of 24. Symptoms persisted for 20.5 days until admission. 48% of IE cases were caused by blood culture-negative.	Males were more likely to develop IE, and the symptoms persisted for over 20 days until admission. The majority cause of IE was culture-negative origins.
33	Thuny et al., 2012 [40]	France	Observational Cohort Study (n = 328)	Excess mortality was significantly higher in the first year after hospital admissions. Females had a higher risk of mortality.	Mortality increased first year after discharge, and mortality overall was more likely in females.
34	Tleyjeh et al., 2005 [30]	Canada	Population-Based Study (n = 102)	The incidence of IE has no significant change, and the most common microorganism that caused IE was *Streptococcus viridans*. No time trends occurred in the rate of 6-month mortality or valve surgery during the study.	No change was observed in the incidence of IE but *Streptococcus viridans *was found to be the most common microorganism.

Discussion

Incidence of IE Between Sexes

The incidence of the development of IE overall as of 2019 was an average of 7.03/100,000 person-years [12, 13]. Males typically have a higher incidence than females regarding the development of IE [12, 14-31]. The incidence rate of IE in males was, on average, 11.39/100,000 person-years; in females, it was 5.24/100,000 person-years [12, 24]. On average, males comprised 65.8% of patients who developed IE, while females comprised only 34.2% of patients [12, 14-19, 21-23, 26, 27, 29-31]. However, one study done in 2022 found that the incidence of developing IE between males and females was equal [32].

It is essential to consider the overall trend in the diagnosis of IE through the years. Between 1990 and 2019, there has been an increasing trend in the development of IE, with one study finding an increase from 6.2/100,000 person-years to 10.2/100,000 person-years [12, 16, 17, 25, 33]. This trend could be due to a growing number of adults with congenital heart disease, an aging population with multiple health issues, shifts in healthcare practices, increased IV drug abuse rates, and improved detection due to advanced diagnostic techniques [33]. Two studies observed that the increasing trend in the development of IE did not vary between the sexes [12, 32]. The remainder of the studies observed that with the growing trend in IE, males had a higher increase in detection than females, and only one study observed this as a non-significant trend [20, 22, 24, 33, 34]. Jensen et al. observed that this was explicitly true concerning prosthetic heart valves [24].

Differences in Age

Along with comparing the incidence of IE between the sexes, it’s also been shown that there is a variation in the age at which each sex develops IE. Most patients with IE were between 60 and 79 years old, with an average of 62.2 years old [17-19, 22, 35, 36]. Erichsen et al. observed that the mean age of IE diagnosis in both sexes has increased steadily, but no study reports variations towards one sex [22]. The actual age differences between sexes were split, but most studies did observe that females were older than males at the diagnosis of IE at the age of 69.3 years old compared to males who were at an average age of 66 years old [12, 22, 23, 26-28, 35-39].

Three other studies observed that females tended to be significantly younger than males [21, 29, 32]. Tariq et al. observed that their patients were significantly younger than in more studies [29]. However, females still presented at a much earlier age than males, with an average age of 16.5 years vs. 34.7 years, but this could be based on the majority type of valve infected by IE reported in this particular study [29]. However, one study found no significant difference in age between the sexes [40]. Bhandari et al. found this trend true when they found that females admitted for drug use-associated IE tended to be significantly younger than males [32]. Despite the age differences in developing IE between sexes, it was found that the overall incidence rates of IE progressively increased with age [12].

Differences in the Valves of IE

The differences in the type of IE stem from what portion of the heart is affected, such as mitral, tricuspid, and aortic valves. This can also be differentiated between native, bioprosthetic, and mechanical valves. Native valve infections occurred on average 71.7% of the time, while bioprosthetic prosthesis valve infections were 17.2%, and mechanical valve infections were 10.7% [17, 19]. The most affected native valve was found to be the mitral valve at 41.7% regardless of the patient's sex, while the least affected valve in general was the pulmonic valve [19]. In assessing which side was affected the most between sexes in general, there was a complete split, with half observing that males were more likely to develop right-sided IE at 40.5%, and the other 75% of males developed left-sided IE [27, 37, 41]. When looking closely at the specific valves, females were more likely to develop IE of the mitral and tricuspid valve whether or not the valve was native or prosthetic [23, 26, 32, 37, 39]. Compared to females, males were more likely to develop IE in their aortic valves, especially native valves, with one study observing up to 73.9% [13, 19, 23, 26, 32, 38, 39, 42]. Notably, the proportion of patients diagnosed with IE involving the native and mechanical valves has decreased. At the same time, there was an increase in the proportion involving bioprosthetic valves in the past decade [17].

Comorbidities

It was discovered that males and females have different comorbidities associated with the development of IE. Considering IE overall, common comorbidities and conditions associated were cardiovascular comorbidities such as congenital heart diseases, hypertension, atrial fibrillation, heart failure, chronic kidney disease, COPD, and diabetes [17, 24, 29, 42]. Becher et al. observed that hypertension is the highest comorbidity associated with patients with a diagnosis of IE at up to 44.9% [17]. In comparing the sexes, four studies observed differences in their Charlson comorbidity index, but many others found differences [20, 26, 35, 40].

Two studies found that females typically had more comorbidities, supported by other studies' findings [12, 14]. Females were more likely to have diabetes, atrial fibrillation, arterial hypertension, hypercholesterolemia, take immunosuppressants, chronic lung disease, malignancy, previous valve replacement, and psychiatric disorders like depression/bipolar disorder [14, 21, 26, 27, 31, 32, 39, 41, 43]. However, when this was crossed with females who had type 2 diabetes mellitus, females specifically had a higher incidence of dementia, atrial fibrillation, previous mitral valve disease, and previous tricuspid valve disease [43]. Compared to females, another study observed that males have a greater rate of comorbidities with a higher rate of chronic liver disease, IE on top of normally structured heart valves, underlying valve disease, peripheral vascular disease, coronary artery disease, ischemic cardiomyopathy, human immunodeficiency virus, previous procedures or intervention, coagulopathy, uncontrolled hypertension, tumors, and in most articles smoking, drinking, and drug use [14, 16, 21, 23, 26, 27, 31, 32, 39, 41, 42]. Specifically, males who developed IE and who also had type 2 diabetes had a higher incidence of chronic lung disease compared to females [43].

With drug, alcohol, and substance use, there was no consensus on which sex was more likely to do either of them. Bhandari et al. observed no difference between substance or alcohol abuse between the sexes [15, 16, 32]. A couple of studies, however, observed that males were more likely to participate in IV substance abuse, smoking, and alcohol abuse [14, 21, 31, 41]. Afshar et al. did explain that due to their population base being in Iran, this result may be skewed as female smoking in Iran is frowned upon [14].

Etiology and Microorganism

IE can develop through many means, and these means are called culprit procedures, which consist of IVs, dialysis, early prosthetic valve endocarditis, non-cardiac surgeries, urinary catheter insertions, and dental procedures [21]. There was no difference in culprit procedures leading to IE [21]. One study evaluated how patients acquire the infection, and one study found that nosocomial IE was similar between the sexes [26]. However, this is contraindicated by Elamragy et al. and Sevilla et al., who found that females were more likely to have nosocomial IE [21, 39].

Common organisms involved in the development of IE are *Staphylococcus aureus, Streptococci spp., Enterococcus*, gram-negative spp., and culture-negative [17, 19, 21, 23, 25, 28-30, 33, 39, 44]. The most common microorganism to lead to IE was inconclusive between studies, with four observing that was *Staphylococcus aureus* up to 43.9% of patients, and this was true in comparing nosocomial and community-acquired IE, followed by *Streptococcus spp*. [17, 19, 21, 23, 25, 33, 39]. Other studies observed that *Streptococci viridans* was the most common microorganism to lead to IE with up to 35% of patients and was followed by *Staphylococcus aureus* [17, 28-30]. Anderson et al. observed that *Enterococcus* IE did not occur as frequently, with only 9.8% of patients affected [44]. Only a tiny percentage of patients had non-specified/atypical bacterial infections, and it was found that these organisms were more likely to cause IE in prosthetic valves [19, 28]. Culture-negative endocarditis, however, was found to be almost as frequent as *S. aureus* infections, with an average of 38.7% of patients being affected [17, 19, 23, 29]. Bezerra et al. observed that negative culture IE had a non-significant trend affecting the mitral valve more than the aortic valve [19].

Assessing between sexes, some studies found no difference in the microorganisms leading to IE, with *Staphylococcus aureus* being the most common [21, 26, 32, 38, 40]. A recent study, however, found that females tended to have more frequent positive blood cultures [31]. The most common microorganisms in females were *Staphylococcus​​​​​​​ aureus, Streptococci spp*., and culture-negative [16, 20, 27, 31, 32, 38, 39, 41]. Some studies observed that *Staphylococcus​​​​​​​ aureus* was the most common microorganism to lead to IE in females, including methicillin-resistant *Staphylococcus​​​​​​​ aureus* [16, 31, 32]. Sevilla et al. observed that coagulase-negative *Staphylococci* was the 2nd most common microorganism in females [39]. Compared to males, most studies observed that females were significantly more likely to have gram-negative IE, with one study proposing that this is due to females suffering from genitourinary infections more frequently [20, 27, 39]. Contrary to the previous statement, Bansal et al. observed that gram-negative bacteria infected more males [16]. Also, females were more likely to be infected with culture-negative IE than males, and at times, even at a higher rate than *Staphylococcus​​​​​​​ aureus* [38, 41]. However, two previous studies found no difference in the incidence of culture-negative IE between the sexes [21, 26].

Contrary to previously mentioned, an earlier study observed that males were more likely to have culture-positive IE [29]. The most common microorganism to affect males were *Staphylococcus​​​​​​​ aureus, Streptococci spp., Enterococcus*, and coagulase-negative *Staphylococci*, which was only slightly different compared to females [15, 16, 20, 27, 31, 32, 38, 39, 41]. *Streptococcus viridans* was the most common microorganism observed to lead to IE in males compared to females, which may be because this microorganism tends to infect native valves, which is the common valve to develop IE in males [16, 20, 27, 32, 38, 39, 41]. The second most common microorganism in males compared to females was *Enterococcus*, but some studies found this as the number one, followed by *Streptococcus viridans* [16, 27, 31, 32, 39, 44]. One study did observe that there was a significantly higher percentage of males who were infected with coagulase-negative *Staphylococcus spp*. compared to females [15].

To better understand these differences and the fluctuation between the predominant microorganism, the trend of these microorganisms need to be considered. *Staphylococcus spp*. has been observed to have a non-significant increasing trend in occurrence, which might explain its predominance [17, 19]. *Streptococcus spp*. however, is on the decline in occurrence, but this finding was non-significant and may be why it is now essentially the second most common microorganism [19]. However, this does not agree with what Becher et al. observed: *Streptococcus spp*. has remained relatively stable over the years [17]. Both Bezerra et al. and Becher et al. observed that blood culture-negative causes of IE have decreased over the last few decades [17, 19]. Studies have yet to agree upon a solid ranking of microorganisms between the sexes, which is why the majority was presented in this format.

Risk Factors

Regarding risk factors for developing IE, they remained relatively similar between males and females. Risk factors for developing IE in either sex were prosthetic valves, having undergone prior valve surgery, implanted pacemakers or defibrillators, and diabetes [13, 25, 44]. Specifically, regarding aortic valve replacement associated with IE, cardiac implantable electronic devices, a bioprosthetic valve, atrial fibrillation, cancer, and diabetes were significant risk factors [25]. Also, in assessing mitral valve replacement associated with IE, the risk factors were bioprosthetic valve and heart failure [25]. In both valve replacements, however, being male was also a significant risk factor in developing IE [25]. IV drug use is also considered a risk factor for developing IE, but most studies found that male patients tended to be the ones to use [14, 21, 31, 41].

With females, specific risk factors for developing IE were type 2 diabetes mellitus, end-stage renal disease with dialysis, and immunosuppression therapy [15, 37]. Specific risk factors for developing IE in males were male, coronary artery bypass graft, coagulopathy, infected cardiac device/implant, previous endocarditis, and left ventricular ejection fraction < 30% [16, 23]. In both sexes, immunosuppressants were a significant risk factor [15, 23]. Ahtela et al. observed that sex differences in risk factors for developing IE increase with age up until 59 years old, decreasing [12].

Manifestations of IE

IE can have many different manifestations and sequential complications. In patients who develop IE, fever was seen in 94% of patients and was considered the number one symptom [29]. Up to 80.7% of patients developed vegetations, which was revealed when an echocardiogram was performed [31]. Common cardiac complications aside from vegetation consisted of advanced heart failure, cardiac valve abscess, and left ventricular failure, seen in up to 36% of patients [21, 29]. Non-cardiac complications that were most common were fulminant sepsis, renal complications such as glomerulonephritis, microscopic hematuria, and renal failure with dialysis, and neurological complications like cerebral embolism, seizures, intracranial hemorrhage, and cerebral abscess [21, 29].

Elamragy et al. concluded that the duration of symptoms of IE before admission was very long but did not differ between sexes [21]. Between the sexes, there was no significant difference in initial symptoms such as fever, skin lesions, systemic embolism, and new onset heart failure [38]. Polishchuk et al. found no significant difference in either echocardiographic findings regarding vegetation or abscesses between sexes [41]. Both sexes showed the development of the same vegetation size, cardiogenic shock incidence, peripheral embolism, heart failure, acute renal failure, stroke, conduction disturbances, and fulminant sepsis [15, 21, 26, 31, 38, 40].

The median symptom duration in females was 30 days at the presentation time [29]. Females with IE were more likely to develop vegetations on intracardiac devices, catheters, and mitral and aortic valves, as well as development of ring abscesses [15, 21, 31]. Elamragy et al. observed that females have significantly higher complication rates, which was confirmed when Sevilla et al. observed that females were more likely to present with septic shock, and Weber et al. found that neurological symptoms occurred more in females [21, 31, 39]. However, Curlier et al. observed the opposite effect, with females significantly less likely to develop septic shock following IE diagnosis [37].

Compared to females, the median symptom duration was 20 days in males at the presentation time [29]. The tricuspid valves were more likely to develop vegetation in males than females [21]. Additionally, males were more likely to have new conduction abnormalities and intracardiac abscesses [15, 26]. Males also present with higher rates of significant valvular regurgitation, which may be because they present with native IE and a greater chance of developing pseudoaneurysms [39]. Possibly due to the pseudoaneurysms, males tend to have more paravalvular complications due to valve perforation [26, 39]. Contrary to Weber et al.’s observation that females tended to have more neurological complications from IE, Sambola et al. observed that stroke complications were more frequent in males [26, 31].

Treatment

Between 2001 and 2020, admissions of IE grew significantly, with a median and mean length of stay being 20 days and 24 days, and tended to be significantly longer in patients who recorded a Charles comorbidity index of 0 [17, 18, 35]. It was observed that gram-negative bacteria seem to have a longer duration of infection as well as longer hospital stays [17]. The initial treatment of IE was antibiotics, primarily penicillin G and gentamicin, to treat the most common microorganisms [29, 44]. Factors associated with lower use of surgery were female sex, current immunosuppressive therapy, and end-stage renal disease with dialysis [15]. However, antibiotics vary greatly between the microorganisms involved to treat them best [29]. For example, patients infected with *Enterococcus avium* and *Enterococcus​​​​​​​ casseliflavus* were treated with vancomycin, streptomycin, gentamicin, or ampicillin [44]. At times, certain microorganisms, such as *Enterococcus faecium*, are resistant to most antibiotics, and alternate treatments, such as surgery, may need to be performed [44]. Factors that predicted the need for surgery were the acute presentation of IE, the development of congestive heart failure, a pacemaker, and an intracardiac abscess [15]. Early valve surgery is indicated by predictors like younger age, valve perforation, aortic location of IE, severe regurgitation, intracardiac abscess, and congestive heart failure [37]. Up to 3.9% of hospitalized patients undergo extraction of previously implanted cardiac devices [17]. Sometimes, a new prosthetic valve, pacemaker generator change, or pacemaker implantation must be done [17, 35]. It is important to know that the number of patients per year receiving surgical treatment for IE has increased between 2002 and 2020 [16, 23].

Comparing the sexes, Panagides et al. found no variation in the duration of the hospitalization between males and females [38]. One study found there was no difference in the treatment strategies of medical and surgical plans between the sexes, which may be backed up by the fact that preoperative characteristics such as surgery risk, heart failure, ejection fraction, cardiac risk factors, and New York Heart Association (NYHA) class were similar [21, 26, 31, 39, 41]. When assessing early valve surgery, Curlier et al. observed no difference between native and prosthetic valves in both sexes [37]. Polishchuk et al. observed no difference in valvular replacement, recurrent valve replacement, and removal of pacemakers/electrodes between sexes [41]. For the patients who did undergo surgery, there was no significant difference in the duration of hospital stay between the sexes [16, 23, 31, 42].

Contrary to Panagides et al., two studies observed that the length of hospital stay was an average of 18.85 days, a shorter duration than males [16, 20, 35, 36, 38]. In fact, females were more likely to be granted an emergency status and an escalation of care [18]. Females, however, were significantly more likely to receive medical treatment first than to undergo surgical intervention [20, 26, 27, 36-38, 40]. When females did undergo surgery, mitral valve replacement was the more commonly performed surgery [31]. Over the past years, there has also been an increase in mitral valve replacement, consistent with the increase in surgical intervention [16].

Regarding males, males had significantly increased hospitalization rates for IE, especially when type 2 diabetes mellitus was present [18, 43]. Also, contrary to Panagides et al., the length of stay was an average of 20.3 days and was mostly considered significantly longer [16, 20, 23, 27, 35, 38]. While males underwent medical management, they were less likely to undergo this type of management than females [26]. On the other hand, males not only developed indications for surgical treatment but were also more likely to undergo surgical intervention, with one study reporting 33.3% of males compared to 20.9% of females [15, 26-28]. Bansal reported that males who underwent surgical intervention were younger and had an increased prevalence of comorbidities and risk factors compared to females [16]. When undergoing surgery, males were more likely to undergo aortic valve replacement than females because the aortic valve is more likely to be infected by males [31, 45]. This surgical intervention has also increased, correlating with the increase in surgical intervention in general [16]. Following surgery, males were more likely to receive blood transfusions and needed longer hospital stays than females [23]. Despite the increased rate of surgical interventions, males were less likely to have an escalation of care. Also, compared to females with type 2 diabetes mellitus in treating IE-associated renal failure, males with type 2 diabetes mellitus were significantly less likely to undergo dialysis [43].

Outcomes

The overall survival of patients with IE was 61%, but when looking at mortality, it’s important to consider the risk factors associated with each mortality period concerning IE. Risk factors for in-hospital mortality were being female, advanced age, heart failure, chronic pulmonary disease, stroke, myocardial infarction, renal failure with dialysis, need for invasive ventilation, a history of diabetes, age, septic pulmonary infarcts, infection with *Staphylococcus aureus*, gram-negative bacteria, intracranial hemorrhage, and persistently positive blood culture [15, 17, 35]. Patients with *Streptococcus* pathogens causing their IE had a smaller risk of in-hospital mortality [17]. In-hospital mortality has increased from 13.4% to 16.5% by 2019, with all common pathogens [17]. Risk factors for 30-day, 1-year, and long-term mortality carried similarities from in-hospital mortality but also included previous cardiac surgery, high left ventricular ejection fraction, low cardiac output, ventilation before the operation, high NYHA class, septic shock, CRP level > 120 mg/L, creatine higher than 180 umol/L, early valve surgery, fungal endocarditis, hepatic cirrhosis, combined mitral and aortic valve replacement, availability of vegetation on transesophageal echocardiography, and presence of a chronic central catheter [15-17, 25, 41, 42]. 30-day mortality rate following IE diagnosis was 11.3%, and 1-year mortality was 22.7% [35]. Compared to the mechanical valve, there is an increased mortality risk in patients with bioprosthetic aortic valve replacement and mitral valve replacement [13]. There was an increase in mortality rate among patients with IE as the age category increased and was agreed upon with the age-standardized mortality rate [12, 19, 25, 34].

Between the sexes, there was no difference in all-cause survival at follow-up, and this trend was similar after surgery [23]. However, two studies observed that surgical treatment increased survival rates and was protective of in-hospital mortality rates in both sexes [15, 26]. There was no difference between the sexes after surgery in neurological complications [42, 45]. Also, CKD and hemodialysis did not differ between sexes following surgical treatment [42]. Even up to 10 years following initial surgical aortic valve replacement as IE repair, there was no difference between sexes on re-surgical intervention [45]. Ultimately, mortality and long-term complications following surgical intervention did not vary between sexes [26, 39].

Several significant predictors of in-hospital mortality in both sexes consist of Charlson comorbidity index > 2, heart failure, dialysis in type 2 diabetes mellitus patients, and strong renal failure [23, 26, 43]. Several studies observed that the in-hospital mortality rate did not differ between the sexes [16, 21, 36-39, 41]. *Streptococcus* was associated with decreased in-hospital mortality in both sexes, but only when patients were stratified by type 2 diabetes mellitus status [43]. When adjusting for type 2 diabetes mellitus, in-hospital mortality was significantly increased in both sexes [43]. Overall, however, in both sexes, older age was associated with a greater in-hospital mortality rate [20]. On the treatment side, early valve surgery was associated with a significantly decreased risk of long-term mortality and an increased risk of short-term mortality in both sexes [37]. Despite this, re-admission in the first month did not vary between sexes [41]. Between both sexes, there has also been a significant decrease in in-hospital mortality rates among patients who underwent valve replacement surgery [16].

Persistent fever, heart failure, acute renal failure, and stroke were significant predictors of 1-year mortality, while surgery was protective variable at 1-year, but 1-year mortality did not differ between sexes [20, 26, 27, 35, 37, 45]. Myllykangas et al. observed this association up to 10 years following IE diagnosis, including major bleeding episodes in the gastrointestinal and intracranial sites following surgical aortic valve replacement [45]. Overall mortality was similar between sexes and age differences in mortality rates [12, 16, 19]. The recurrence rate did not vary between sexes [38, 41]. The trends in overall mortality have varied but were similar between sexes in each study, with one stating that there was a steady decline in mortality rates [16]. The other two studies also offered opposite results, stating no trend being observed and the other noting an increase in mortality [19, 34].

In comparing females to males, females were significantly more likely to leave against medical advice, almost twice as males, and experienced a survival rate of 72% for in-hospital stay and 81% and 62% at 1 year following diagnosis, which started with being better than males [23, 26, 32]. Regarding the in-hospital mortality rate, important predictors were poor response to antibiotics, heart failure, septic shock, surgery, type 2 diabetes mellitus, and persistent bacteremia [16, 21, 38, 43]. Multiple studies found that females had an increase in in-hospital mortality; however, this was less than those studies stating that there was no significant difference between sexes [15, 20, 23, 26, 38]. However, one study found that females had a lower in-hospital mortality rate than males [27]. As mentioned, there’s a decrease in in-hospital mortality rates following valve replacement surgery; however, the reduction is greater in females according to one study [16]. Concerning the 30-day mortality, females were an independent predictor, preoperative transfer from intensive care units, and increased C-reactive protein [23]. 30-mortality was also significantly increased in females compared to males and was associated with increased age, endocarditis focus identification, preoperative dialysis, and peripheral arterial disease [23, 31, 42].

Regarding the 1-year mortality, female sex was found to be an independent predictor, which correlates with studies reporting that 1-year mortality was significantly increased in females [26, 31, 41]. While one study found a decreased risk of death amongst females with IE, five other studies found that females had a significantly greater mortality rate [14, 18, 25, 36, 40]. Predictors of overall mortality in females had some similarities with overall IE patients. Still, they were specifically mentioned to be having aortic valve endocarditis, endocarditis recurrence, *Staphylococcus epidermidis*, coronary vessel disease NYHA IV, emergency admission, diagnosis until surgery > 7 days, septic arterial embolism, previous mitral disease, atrial fibrillation, acute renal disease, diabetes, and heart valve surgery [20, 23, 38, 40]. There was a trend towards increased mortality in females who received elective surgery as they developed acute kidney injury, Staphylococcal bacteremia, coagulopathy, uncontrolled hypertension, malnutrition, chronic rheumatic heart disease, increased rate of ischemic stroke and cerebrovascular events, tracheostomy, and use of mechanical ventilation during the hospital stay [16, 27, 31, 37, 39]. Bhandari et al. observed a significant difference in mortality in older females compared to younger females [32]. Compared to previous studies, two studies found that female sex was not an independent predictor for 30-day, 1-year, and late mortality [23, 31]. Of the females that died, they were more likely to have died from congestive heart failure than males [26]. Overall, there has been a rise in mortality from IE over the last two decades, which was more notable in females [34].

Compared to females, males were significantly more likely to be discharged after treatment completion, with 30-day, 1-year, and 5-year survival rates of 85%, 74%, and 82% [23, 26, 32]. This survival was seen following surgical treatment for aortic valve associated-IE but did not persist after ten years [42]. However, it’s important to note that there was a trend in long-term benefits of early valve surgery in males [37]. Regarding in-hospital mortality, significant predictors for males specifically were poor response to antibiotics, performing surgery when indicated, fulminant sepsis, cardiogenic shock, arterial hypertension, left ventricular ejection fraction < 30%, peripheral artery disease, combined valve surgery, preoperative stroke, tumor, liver disease, abscess, pulmonary hypertension, BMI, fever until surgery, culture-negative IE, abscess, and embolization of several organs [21, 23]. In-hospital complications such as acute renal failure, sepsis, hemorrhagic stroke, and requiring pacemakers were seen more in males [27, 43]. In several studies, males had significantly lower in-hospital mortality rates; however, *Staphylococcus* bacteremia was linked to elevated in-hospital mortality in males [15, 20, 23, 26, 27]. While there has been an increase in in-hospital mortality rates between the sexes, males were observed to have a reduced increase compared to males [16].

When looking at the 30-day mortality rate in males, there was a significant association with age, prior cardiac surgery, insulin-dependent diabetes, preoperative preserved ejection fraction, kissing mitral valve endocarditis, pulmonary hypertension, multi-organ embolization, surgery performed <7 days of starting antibiotics, culture-negative IE, and undergoing combined surgeries [23, 42]. All studies assessing 30-day mortality found that males had significantly lower rates than females [23, 31, 42]. However, males had a significantly higher death rate when hospitalized than females, according to Chew et al. [36].

Cost

Two studies observed the cost differences associated with the sex infected with IE. Females tended to have a lower hospital bill overall because they received less surgical treatment, with an average cost of $3,035 [36]. Compared to another study, the mean hospital charges were more outstanding amongst males, totaling $88,409, compared to $71,196 in females [16].

Limitations

Some limitations of this review revolved around the limited amount of studies that focused solely on the sex differences of IE. Most of the search involved going through many articles that merely mention the sex differences for each component in this paper. While valuable and even significant information could be used, studies focusing on the differences throughout the process of IE from symptom start to outcomes and complications from treatment in an institutional review would reveal more ideally consistent results over the differences.

## Conclusions

Sex disparities are a growing concern in the lifelong use of healthcare. Like any other condition, there is a difference between the sexes regarding incidence, age of occurrence, etiology, risk factors, manifestations, treatment, outcomes, and cost concerning infective endocarditis (IE). Males were significantly more likely than females to develop IE, tended to be young, and were more likely to have their native aortic valves involved. Concerning comorbidities, females were more likely to have diabetes, atrial fibrillation, arterial hypertension, take immunosuppressants, and psychiatric disorders. The etiology of IE did not vary much between the sexes as the origin usually stemmed from the same culprit procedures, and *Staphylococcus aureus* was the most common organism. Females were more likely to develop IE from culture-negative bacteria than men usually infected with *Streptococcus viridans*. Common risk factors in females included end-stage renal disease, type 2 diabetes mellitus, and immunosuppressive therapy, while with males, it was male, coronary artery bypass graft, coagulopathy, and left ventricular ejection fraction < 30%. Manifestations in females endured for a median of 30 days until admission, and they tended to develop vegetations on catheters, intracardiac devices, and ring abscesses. Females had a longer hospital stay, and they were more likely to receive just antibiotic treatment. Females were significantly more likely to leave against medical advice, possibly contributing to a slight increase in in-hospital mortality. Thirty-day and 1-year mortality was significantly increased in females, with a significant difference in mortality between younger and older females.

Sex differences in health is a topic that is still being acknowledged today, mainly because it’s already known that no specific gold treatment treats everyone the same way. Studies between racial and ethnic variations have already been well underway as researchers have observed that particular drugs work better in different races, such as African Americans being treated more successfully for hypertension with calcium channel blockers compared to inhibitors of the renin-angiotensin-aldosterone system. Treating sex differences requires a multidisciplinary approach, as environmental and biological factors greatly influence whether or not one goes to the hospital for treatment and how one recovers. This study aimed to elucidate the sex differences in IE as mortality rates are in IE. The mortality rates can be further addressed by tailoring treatments appropriate towards the different sexes or making physicians aware of their different approaches towards the sexes. This is shown by females being less likely to undergo surgical treatment, and at the same time, they tend to have a higher long-term mortality. Future research should focus on a couple of things: factors that influence the microorganisms involved in IE between sexes. These aspects of comorbidities affect the development of IE between sexes and, most importantly, evaluating and assessing factors that influence the treatment of IE.
